# Alzheimer's disease and the microbiome

**DOI:** 10.3389/fncel.2013.00153

**Published:** 2013-09-17

**Authors:** Surjyadipta Bhattacharjee, Walter J. Lukiw

**Affiliations:** Departments of Neurology, Neuroscience and Ophthalmology, LSU Neuroscience Center, Louisiana State University Health Sciences CenterNew Orleans, LA, USA

**Keywords:** Alzheimer's disease, evolution, gastrointestinal, innate immune response and inflammation, Metchnikoff, neuro-inflammation, probiotics, symbiosis

“Microbial colonization of mammals is an evolution-driven process that modulates host physiology, many of which are associated with immunity and nutrient intake”—Heijtz et al.(2011)

The recognition of the human microbiome (HM) as a substantial contributor to nutrition, health and disease is a relatively recent one, and currently, peer-reviewed studies linking alterations in microbiota to the etiopathology of human disease are few. Emerging studies indicate that the HM may contribute to the regulation of multiple neuro-chemical and neuro-metabolic pathways through a complex series of highly interactive and symbiotic host-microbiome signaling systems that mechanistically interconnect the gastrointestinal (GI) tract, skin, liver, and other organs with the central nervous system (CNS). For example, the human GI tract, containing 95% of the HM, harbors a genetically diverse microbial population that plays major roles in nutrition, digestion, neurotrophism, inflammation, growth, immunity and protection against foreign pathogens (Forsythe et al., 2012; Collins et al., 2013; Douglas-Escobar et al., 2013; see below). It has been estimated that about 100 trillion bacteria from up to 1000 distinct bacterial species co-inhabit the human GI tract, albeit in different stoichiometries amongst individuals, and the varying combinations and strains of bacterial species amongst human populations might contribute, in part, to “***human-biochemical***” or “***genetic-individuality***” and resistance to disease (Aziz et al., 2013; Lukiw, 2013). Interestingly, HM participation in human physiology may also help explain the genome-complexity conundrum—for example why the 26,600 protein-encoding transcripts in *Homo sapiens* are far fewer in number, than for example, the rice genome (*Oryza sativa*; which has about 46,000 functional genes). One thousand different strains of bacteria might be expected to contribute up to 4 × 10^6^ potential mRNAs to the human transcriptome, thus making the human host-plus-microbiome genetic complexity closer to 4,026,600 mRNA transcripts, and a clear “winner” of human genetic complexity over that of rice and other species (Venter et al., 2001; Foster and McVey Neufeld, 2013; Lukiw, 2013). The very recent observation of microbiome-derived small non-coding RNA (sncRNA) and micro RNA (miRNA) translocation and signaling across endothelial barriers, between cells and tissues, and even perhaps between individual species indicates that human neurobiology may be significantly impacted by the actions of HM-mediated sncRNA or miRNA trafficking, and the integration of a cell, tissue or an entire organism into its local environment (Zhao et al., 2006; Alexandrov et al., 2012; Sarkies and Miska, 2013; Reijerkerk et al., 2013; unpublished). This opinion paper encompasses what we know concerning the contribution of the HM to neurological disease, with specific emphasis on Alzheimer's disease (AD) wherever possible.

Firstly, the microbiome of the human GI tract is the largest reservoir of microbes in the body, containing about 10^14^ microorganisms; over 99% of microbiota in the gut are anaerobic bacteria, with fungi, protozoa, archaebacteria and other microorganisms making up the remainder. There is currently an expanding interest in the ability of intestinal bacteria to influence neuro-immune functions well beyond the GI tract. Since mitochondria are believed to originate from bacteria via endosymbiotic relationships that formed very early in the evolutionary history of eukaryotes, cross-reactivity of mitochondria and immunological responses to intestinal bacterial constituents could have deleterious effects on mitochondrial function through some form of molecular mimicry; this is partially evidenced by the inflammatory basal ganglia disorder Sydenham's chorea, rheumatic fever and the link to the facultative anaerobe *Streptococcus* (Carrasco-Pozo et al., 2012; Douglas-Escobar et al., 2013; Hayashi, 2013; Hornig, 2013 see below). Established pathways of GI-CNS communication currently include the autonomic nervous system (ANS), the enteric nervous system (ENS), the neuroendocrine system, and the immune system (Camfield et al., 2011; Heijtz et al., 2011; Forsythe et al., 2012; Aziz et al., 2013; Collins et al., 2013; Foster and McVey Neufeld, 2013; Schwartz and Boles, 2013). Stress further influences the composition of the HM, and reciprocal communication between the CNS and the HM also influences stress reactivity (Forsythe et al., 2012; Foster and McVey Neufeld, 2013). Surprisingly, neuronal signaling pathways along the bidirectional GI-CNS axis remain poorly understood despite their important roles: (i) in coordinating metabolic- and nutritive-functions, and (ii) in their functional disruption in chronic diseases such as metabolic syndrome, diabetes, obesity, anxiety, autoimmune-disease and stress-induced neuropsychiatric disease (Lukiw and Bazan, 2006; Bravo et al., 2012; Foster and McVey Neufeld, 2013; Hornig, 2013; Udit and Gautron, 2013). Studies of the ENS in “germ-free” mice, i.e., those missing their microbiome, indicates that commensal intestinal microbiota are absolutely essential for passive membrane characteristics, action potentials within the ENS, and the excitability of sensory neurons, thus providing a potential mechanistic link for the initial exchange of signaling information between the GI tract microbiome and the CNS (Foster and McVey Neufeld, 2013; Hornig, 2013; McVey Neufeld et al., 2013). Indeed, secretory products of the GI microbiome and translocation of these signaling molecules via the lymphatic and systemic circulation throughout the CNS are just beginning to be identified. For instance, the GI tract-abundant gram-positive facultative anaerobic or microaerophilic *Lactobacillus*, and other *Bifidobacterium* species, are capable of metabolizing glutamate to produce gamma-amino butyric acid (GABA), the major inhibitory neurotransmitter in the CNS; dysfunctions in GABA-signaling are linked to anxiety, depression, defects in synaptogenesis, and cognitive impairment including AD (Aziz et al., 2013; Hornig, 2013; Mitew et al., 2013; Paula-Lima et al., 2013; Saulnier et al., 2013). To cite another important example, brain-derived neurotrophic factor (BDNF) has pleiotropic effects on neuronal development, differentiation, synaptogenesis and the synaptic plasticity that underlies circuit formation and cognitive function, and has been found to be decreased in brains and serum from patients with schizophrenia, anxiety and AD (Carlino et al., 2013; Lu et al., 2013; Mitew et al., 2013). In experimental infection models known to lead to alterations in the microbiota profile, BDNF expression was found to be reduced in the hippocampus and cortex of “germ free” mice, and this reduction in the expression of BDNF was found to associate with increased anxiety behavior and progressive cognitive dysfunction (Carlino et al., 2013; Foster and McVey Neufeld, 2013; Lu et al., 2013).

Equally interesting are microbiome interactions with the N-methyl-D-aspartate (NMDA) glutamate receptor, a prominent CNS device that regulates synaptic plasticity and cognition (Lakhan et al., 2013). For example, the NMDA-, glutamate-targeting, glutathione-depleting and oxidative-stress-inducing neurotoxin β-N-methylamino-L-alanine (BMAA), found elevated in the brains of patients with amyotrophic-lateral sclerosis (ALS), Parkinson-dementia (PD) complex of Guam and AD, has been hypothesized to be generated by cyanobacteria of the intestinal microbiome, and stress, GI tract disease or malnutrition may further induce BMAA abundance to ultimately contribute to neurological dysfunction (Brenner, 2013). Other HM-resident cyanobacteria-generated neurotoxins such as saxitoxin and anatoxin-α may further contribute to human neurological disease, especially during aging when the intestinal epithelial barrier of the GI tract becomes more permeable (Tran and Greenwood-Van Meerveld, 2013). Interestingly, BMAA, a neurotoxic amino acid not normally incorporated into protein, has been linked with intra-neuronal protein misfolding, a hallmark feature of the amyloid peptide-enriched senile plaque lesions, and resultant inflammatory neurodegeneration, that characterize PD, AD and prion disease (He and Balling, 2013; Hornig, 2013; Mulligan and Chakrabartty, 2013; Schwartz and Boles, 2013). Hence, besides potentially altering CNS neurochemistry and neurotransmission, HM-bacteria not only secrete molecules that potentially modulate systemic- and CNS-amyloidosis, they also widely utilize their own amyloid peptides as structural materials, adhesion molecules, toxins, molecules that function in the protection against host defenses and auto-immunity. The specific contribution of host bacteria and bacterial amyloid, however, to misfolding, amyloidogenic diseases such as AD remain to be more clearly defined (Schwartz and Boles, 2013). The HM further appears to condition host immunity to foreign microbes, including viral infection and xenobiotics, while regulating autoimmune responses that can impact homeostatic metabolic- and neural-signaling functions within the CNS (Ball et al., 2013; Douglas-Escobar et al., 2013; Hornig, 2013). An increased prevalence of autoimmunity, exposure to pathogens both pre- and post-natally, and findings of anti-brain antibodies, common in disorders as diverse as anxiety, schizophrenia, obsessive-compulsive disorder, depression and autism, together suggest that differences in exposure and genetic vulnerability toward HM-mediated autoimmunity may be significant determinants of age-related neurological disease course and outcome (Ball et al., 2013; Douglas-Escobar et al., 2013; Hornig, 2013).

Regarding potentially pathogenic micobiota stationed outside of the GI tract, about 95% of all humans harbor the highly neurotrophic herpes simplex-1 (HSV-1) in their trigeminal ganglia, but whether this is a neutral or symbiotic relationship, or detrimental to the host, remains open to speculation. Induction of HSV-1, and other forms of endogenous viral reactivation are certainly stress-related, but whether GI tract HM-derived metabolites are involved in these kinds of pathogenic activation mechanisms is not well understood (Hill et al., 2009; Prasad et al., 2012). Recent studies suggest that activation of endogenous HSV-1 or other neurotrophic microorganisms, including host-embedded prions, are intimately linked to neurological stressors linked to amyloidogensis, inflammatory neurodegeneration and progressive cognitive impairment, and may be a contributor to the early development of, or predisposition to, schizophrenia and AD (Hill et al., 2009; Prasad et al., 2012; Ball et al., 2013; Manuelidis, 2013). Indeed, correlation of metabolic and neurological phenotypes with the GI tract HM and other specific endogenous bacterial or viral profiles derived from independent molecular analytical technologies should be increasingly useful for deciphering complex host-microbiome relationships in healthy human brain aging and in neuropsychiatric disease (Xie et al., 2013).

Further studies of symbiotic HM-CNS communication intrinsically suggests the potential for microbial-based therapeutic strategies that may aid in the augmentation of the HM, for the treatment of human disease, including neurological disorders (Forsythe et al., 2012; Collins et al., 2013). The original observation of the health-promoting benefits of GI tract bacteria and the HM was first introduced in 1907 by the Russian biologist Ilya Metchnikoff (Nobel Prize in Medicine 1908, shared with Paul Erlich; Buryachkovskaya et al., 2013). Metchnifoff's works focused on prokarytoic immunology, phagocytosis, the anti-aging properties instilled by host bacteria, inflammation as a protective adaptation against injury, and early ideas on neurogastroenterology (Buryachkovskaya et al., 2013; Saulnier et al., 2013). Hence, for well over 100 years, host-beneficial GI tract bacteria, collectively known as probiotics, have been proposed to be useful to human health, and more recently have been added to various foods and diets because of their positive health-promoting effects (Singh et al., 2013). The beneficial actions of bacterial-based probiotics are highly inter-related, and are thought to function, in part: (i) to aid in complex carbohydrate fermentation and absorption; (ii) to provide a significant source of a range of essential vitamins, particularly those of the vitamin B and K group; (iii) to compete with pathogenic microorganisms in the GI tract; (iv) to antagonize and neutralize enteric pathogens; (v) to metabolize and neutralize dietary carcinogens; and (vi) to favorably modulate the host's immune response to resist infection and disease. Besides the potential application of probiotics in the prevention and treatment of various health conditions and diseases such as allergies, GI and urogenital tract infections, inflammatory disease, cystic-fibrosis and certain cancers there is increasing interest of positive microbiome effects toward the CNS via neural, neuroendocrine, neuroimmune and humoral links (Duncan and Flint, 2013; He and Balling, 2013; Saulnier et al., 2013). For example, there is preliminary research on the influence of probiotics and nutritional factors on the prognosis of multiple sclerosis (von Geldern and Mowry, 2012), cognition (Camfield et al., 2011), neurogastroenterology in general (Saulnier et al., 2013), and stress-related psychiatric conditions including anxiety, autism, depression and schizophrenia (Bravo et al., 2012; Prasad et al., 2012; Douglas-Escobar et al., 2013). Advances in probiotic technologies in CNS disease research are already raising a number of ethical, legal, and socioeconomic concerns (Slashinski et al., 2012).

Lastly, the US NIH “Roadmap” program has recently initiated the HM project (HMP; http://commonfund.nih.gov/Hmp/), using recently discovered genomic technologies with the specific aims (i) to characterize the microbial communities at several different sites on the human body, including nasal, oral and otic cavities, the skin, GI and urogenital tracts; (ii) to analyze the role of these microbes in homeostatic human physiology; (iii) to catalog specific microbiome speciation, composition and correlation with disease; and (iv) to generate resources enabling comprehensive characterization of the HM by multiple independent research groups. These investigations present a highly significant and exciting avenue for future study, and suggest new and perhaps unconventional directions for AD research in 2013 and beyond. Future AD therapies may well, in part, involve probiotic approaches, especially as a prophylactic tactic before mild-cognitive impairment (MCI) or AD is first diagnosed. The implications of altered host-HM interactions in neurological disease would be far-reaching indeed, and these may engender novel microbiome manipulative strategies, tailored to the host, for the more effective therapeutic management of AD and related neuropsychiatric disorders.
